# Draft Genome Sequence of the Endophytic *Bacillus aryabhattai* Strain SQU-R12, Identified from *Phoenix dactylifera* L. Roots

**DOI:** 10.1128/genomeA.00718-17

**Published:** 2017-08-10

**Authors:** Mahmoud W. Yaish

**Affiliations:** Department of Biology, College of Science, Sultan Qaboos University, Muscat, Oman

## Abstract

*Bacillus aryabhattai* strain SQU-R12 was isolated from date palm seedlings, where it showed a growth-promoting capacity by being able to synthesize indole-3-acetic acid phytohormone and reduce ethylene biosynthesis by producing 1-aminocyclopropane-1-carboxylic acid deaminase. The draft genome sequence of this strain is reported here.

## GENOME ANNOUNCEMENT

Endophytes have the ability to promote plant growth and development through the production of hormones and mineral solubilization substances (1). In addition, some of these endophytes are able to synthesize an ethylene repression enzyme, 1-aminocyclopropane-1-carboxylic acid (ACC) deaminase, thereby enhancing the plants’ tolerance of various environmental stresses (2). Different groups of endophytes were previously identified from the roots of date palm seedlings (3–5), as well as from other plant species (6). In this work, a draft genome sequence of *Bacillus aryabhattai* strain SQU-R12 was generated in order to uncover the underlying growth-promoting mechanism of this strain, which was previously identified based on functional and biochemical characterizations (4).

The genome was sequenced using the Illumina HiSeq2500 system and the DNA paired-end library method. The sequencing procedure was performed in the DNA sequencing facilities at BaseClear BV (Netherlands). Genes within scaffolds were identified using Glimmer (7), and the genes were annotated using Blast2GO4.1 (8) and the NCBI Prokaryotic Genome Annotation Pipeline.

The DNA sequencing produced 6,310,670 total reads, including 6,231,215 mapped reads in pairs. The assembly results showed that the genome consisted of 5,584,636 bp, assembled into 118 scaffolds ranging in length from 319 to 1,177,917 bp, with an average length of 47,327 bp and a GC content of 37.74%. The genome included 5,635 putative coding sequences and 91 pseudogenes. There were 4,931 genes with a known function, representing 1,552 enzymes localized on the 639 Kyoto Encyclopedia of Genes and Genomes pathways. The list also contained 88 RNA genes, including 5 complete rRNA genes and 75 tRNAs.

The reported strain was previously identified as belonging to the species *Bacillus oleronius* based on a partial 16S rRNA gene sequence (4); however, the near-complete genome sequence revealed that the number of top BLAST hits for the genes was similar to that for the species *B. aryabhattai*. To confirm this result, the genome was compared with the genomes of the type strains and proxytype strains that are already in GenBank using average nucleotide identity as previously described (9). The results showed that this genome is 96.6% identical to *B. aryabhattai* (85.1% coverage of the submitted genome sequence); therefore, this strain was designated *B. aryabhattai* SQU-R12.

The endophytic bacterial genome reported in this project encoded a nitrogen fixation protein (VnfA) and nitroreductase proteins (10); tryptophan synthase alpha and beta chains, which may involve IAA synthesis in bacteria (11); an ACC deaminase/d-cysteine desulfhydrase (12); several putative siderophore biosynthesis, binding, and transport proteins (13); and five ampicillin-resistant beta-lactamase proteins (14). In addition, analysis of secondary metabolite biosynthesis gene clusters using antiSMASH version 3.0 (15) revealed the presence of locillomycin, asukamycin, and iturin antibiotic and biosynthetic gene clusters. Together, the presence of these genes within the genome may enhance the environment–plant microbe interactions and symbiosis.

### Accession number(s).

This whole genome shotgun project was deposited at DDBJ/ENA/GenBank under the accession number NHZZ00000000. The version described in this paper is the first version, NHZZ01000000.
